# Podocalyxin promotes the formation of compact and chemoresistant cancer spheroids in high grade serous carcinoma

**DOI:** 10.1038/s41598-024-57053-7

**Published:** 2024-03-30

**Authors:** Ngoc Le Tran, Yao Wang, Maree Bilandzic, Andrew Stephens, Guiying Nie

**Affiliations:** 1 Implantation and Pregnancy Research Laboratory, School of Health and Biomedical Sciences, RMIT University, Bundoora West Campus, Bundoora, VIC 3083 Australia; 2 Hudson Institute of Medical Research, Clayton, VIC 3168 Australia; 3 Department of Molecular and Translational Sciences, Monash University, Clayton, VIC 3168 Australia

**Keywords:** Cancer models, Gynaecological cancer, Cell death, Cell growth, Biomarkers, Cancer

## Abstract

High grade serous carcinoma (HGSC) metastasises primarily intraperitoneally via cancer spheroids. Podocalyxin (PODXL), an anti-adhesive transmembrane protein, has been reported to promote cancer survival against chemotherapy, however its role in HGSC chemoresistance is unclear. This study investigated whether PODXL plays a role in promoting chemoresistance of HGSC spheroids. We first showed that PODXL was expressed variably in HGSC patient tissues (n = 17) as well as in ovarian cancer cell lines (n = 28) that are more likely categorised as HGSC. We next demonstrated that PODXL-knockout (KO) cells proliferated more slowly, formed less compact spheroids and were more fragile than control cells. Furthermore, when treated with carboplatin and examined for post-treatment recovery, PODXL-KO spheroids showed significantly poorer cell viability, lower number of live cells, and less Ki-67 staining than controls. A similar trend was also observed in ascites-derived primary HGSC cells (n = 6)—spheroids expressing lower PODXL formed looser spheroids, were more vulnerable to fragmentation and more sensitive to carboplatin than spheroids with higher PODXL. Our studies thus suggests that PODXL plays an important role in promoting the formation of compact/hardy HGSC spheroids which are more resilient to chemotherapy drugs; these characteristics may contribute to the chemoresistant nature of HGSC.

## Introduction

Ovarian cancer represents the most lethal gynaecological malignancies worldwide, with more than 300,000 new cases being diagnosed each year^1^. More than 95% of all ovarian cancer are epithelial in origin with HGSC accounting for approximately 70% of all cases^2,3^. Due to the absence of early warning symptoms and the lack of early diagnosis, women with ovarian cancer are typically diagnosed at advanced metastatic stages where the 5-year survival rate is approximately only 30%^4,5^. While patients normally respond well to initial cytoreductive surgery and adjuvant chemotherapy, almost all patients relapse due to chemoresistance^6^; unfortunately, such devastating outcomes have not been improved over the past three decades.

HGSC is unusual in that it rarely metastasises through the circulatory system, which is the driving mechanism for most tumour types. Instead, HGSC often spreads intraperitoneally. This unique mode of metastasis involves exfoliation of cancer cells from primary tumours and the subsequent dissemination of free floating cancer cells into the peritoneal fluid (ascites)^7^. Studies show that these cancer cells rarely exist as single cells, rather they often form non-adherent multicellular aggregates of cancer spheroids, which can be seen floating in ascites or embedded to the mesothelium of the peritoneal cavity during the debulking surgery^8,9^. Ascites represents a dynamic reservoir of cancer spheroids as well as cancer survival factors such as growth factors and tumour-promoting cytokines^7^. Ascites is present in almost all cases of HGSC relapse, and greater volumes are associated with significant morbidities^10–12^. Following chemotherapy, residual cancer cells at the primary tumor sites, together with cancer spheroids floating in ascites, are known to contribute to cancer relapse^13,14^, however, molecular mechanisms are not totally understood.

Past studies of HGSC often utilised monolayer culture which has now been recognised to be of less physiological relevance^15^. This led to the recent rise in the use of 3D culture of cancer spheroids, which are believed to better recapitulate the physiological characteristics of HGSC cells existing within the non-adherent environment of ascites^15,16^. Thus, it is important to use spheroids as a 3D model to study HGSC biology including cancer cell–cell interactions and chemoresistance^17,18^.

PODXL is a transmembrane protein of the CD3 family of sialomucins, and it is normally expressed in kidney glomeruli, vascular endothelia, certain epithelial cells, and hematopoietic progenitor cells^19–22^. However, PODXL is overexpressed in several epithelial cancers including pancreatic, breast and ovarian cancer; and importantly this overexpression has been associated with poor prognosis^23–26^. While the exact mechanisms by which PODXL promotes tumorigenesis is unknown, PODXL is suggested to contribute to budding of non-adhesive metastatic tumour nodules of ovarian and breast cancer^26,27^. Furthermore, suppression of PODXL has been found to substantially reduce astrocytoma and osteosarcoma cancer cell viability and survival against their respective chemotherapy drugs temozolomide and cisplatin^28,29^. For ovarian cancer, PODXL is more highly expressed in HGSC (87%) compared to other subtypes, and PODXL cell surface localisation is reported to be significantly associated with poorer disease-free survival^26^. However, it is unclear whether PODXL plays a causal role in chemoresistance of HGSC.

In this study, utilizing 3D culture of cancer spheroids, we aimed to investigate the functional importance of PODXL in HGSC spheroid including response to chemotherapy drugs. We first examined PODXL expression in HGSC patient tissues (n = 17) by immunohistochemistry, then scrutinized 37 ovarian cancer cell lines for their PODXL expression and analysed the association between the levels of PODXL and the likelihood of being categorised as HGSC. Next, the cell line expressing the highest level of PODXL was used as a model, and cancer spheroid characteristics were investigated by silencing PODXL via CRISPR/Cas9 gene editing. The key features observed in cell lines were then examined in primary cancer cells derived from ascites of HGSC patients (n = 6). Overall, our studies provide in vitro evidence that PODXL promotes the formation of compact and chemoresistant HGSC spheroids.

## Results

### PODXL is immunostained in all HGSC patient tissues with variable intensity

**Figure 1:**
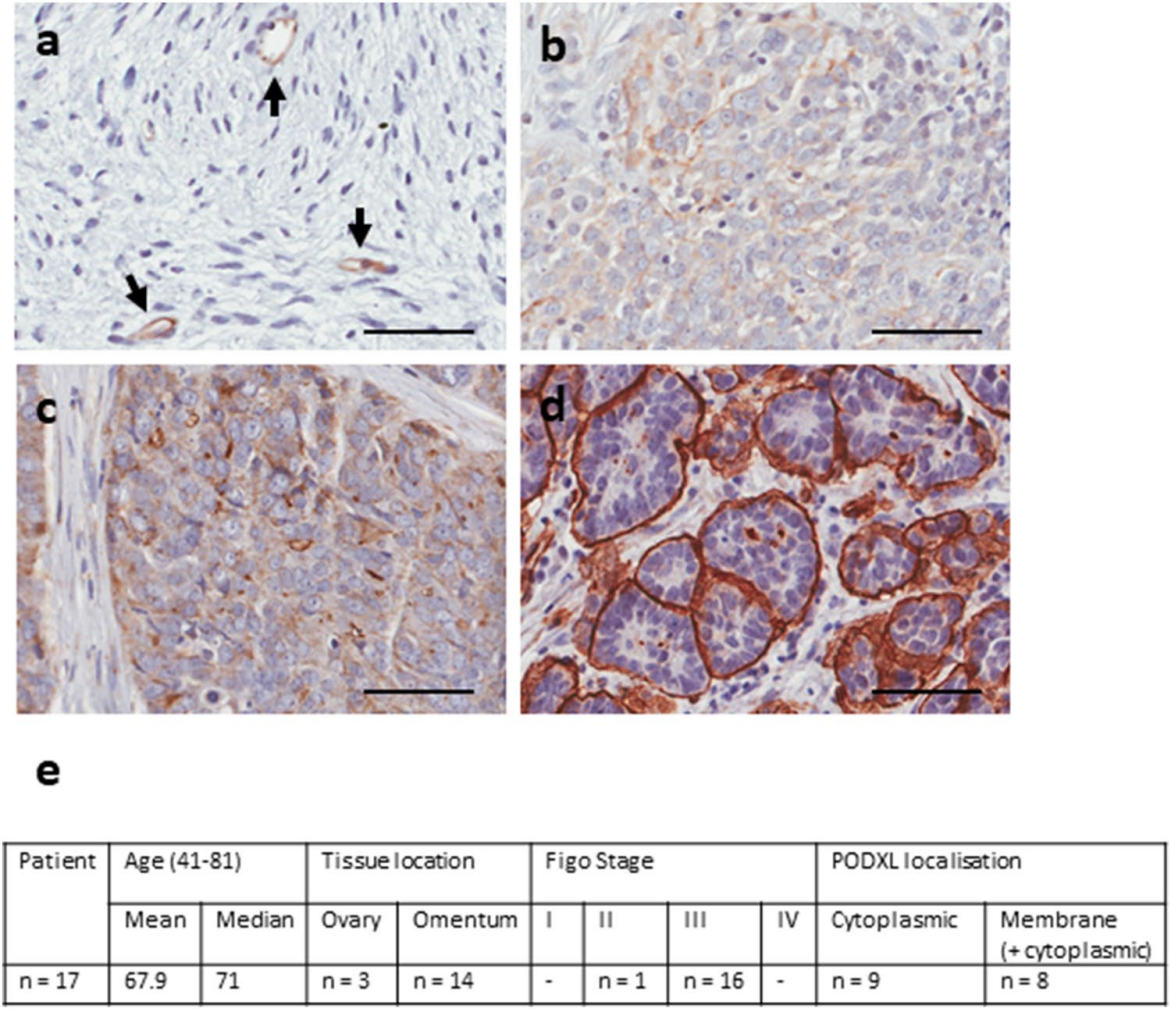
Immunostaining of PODXL in primary tumor biopsies of HGSC patients. Representative images at 16× magnification. Scale bar: 50 µm. (**A**) Benign ovarian tumour. Black arrows, blood vessels. (**B**–**D**) HGSC tumours with weak (**B**), moderate (**C**), and high (**D**) staining intensity of PODXL. (**E**) Summary of clinicopathological characteristics of the cohort examined.

Primary tumor tissues of HGSC patients (n = 17) and one benign ovarian sample were analysed by immunohistochemistry for PODXL. In the benign tissue, PODXL stained only in endothelial cells of blood vessels (Fig. 1A), which are known to express PODXL^26,30^; no other cells were positive for PODXL. In contrast, all HGSC tissues (n = 17) were positive for PODXL with variable staining intensity. Among them, 41% tissues presented low, 29% moderate, and 29% high levels of PODXL immunostaining (Fig. 1B–D). The cellular localisation of PODXL also varied, ranging from cytoplasmic to a mixture of membranous/cytoplasmic and to strongly membranous (Fig. 1E). In general, tissues with low or moderate PODXL staining tended to show cytoplasmic or membranous/cytoplasmic localisation (Fig. 1B,C). In contrast, tissues with high levels of PODXL displayed strong membrane staining (Fig. 1D); furthermore, when clusters of cancer cells were present, PODXL was stained strongly on the outer layer whereas cells within the centre were negative (Fig. 1D). Since over 90% of the samples examined were from patients at FIGO stage III (Fig. 1E), no clear correlation could be ascertained between PODXL intensity and disease severity.

### PODXL is expressed in many ovarian cancer cell lines and those with higher expression are more likely categorised as HGSC

**Figure 2:**
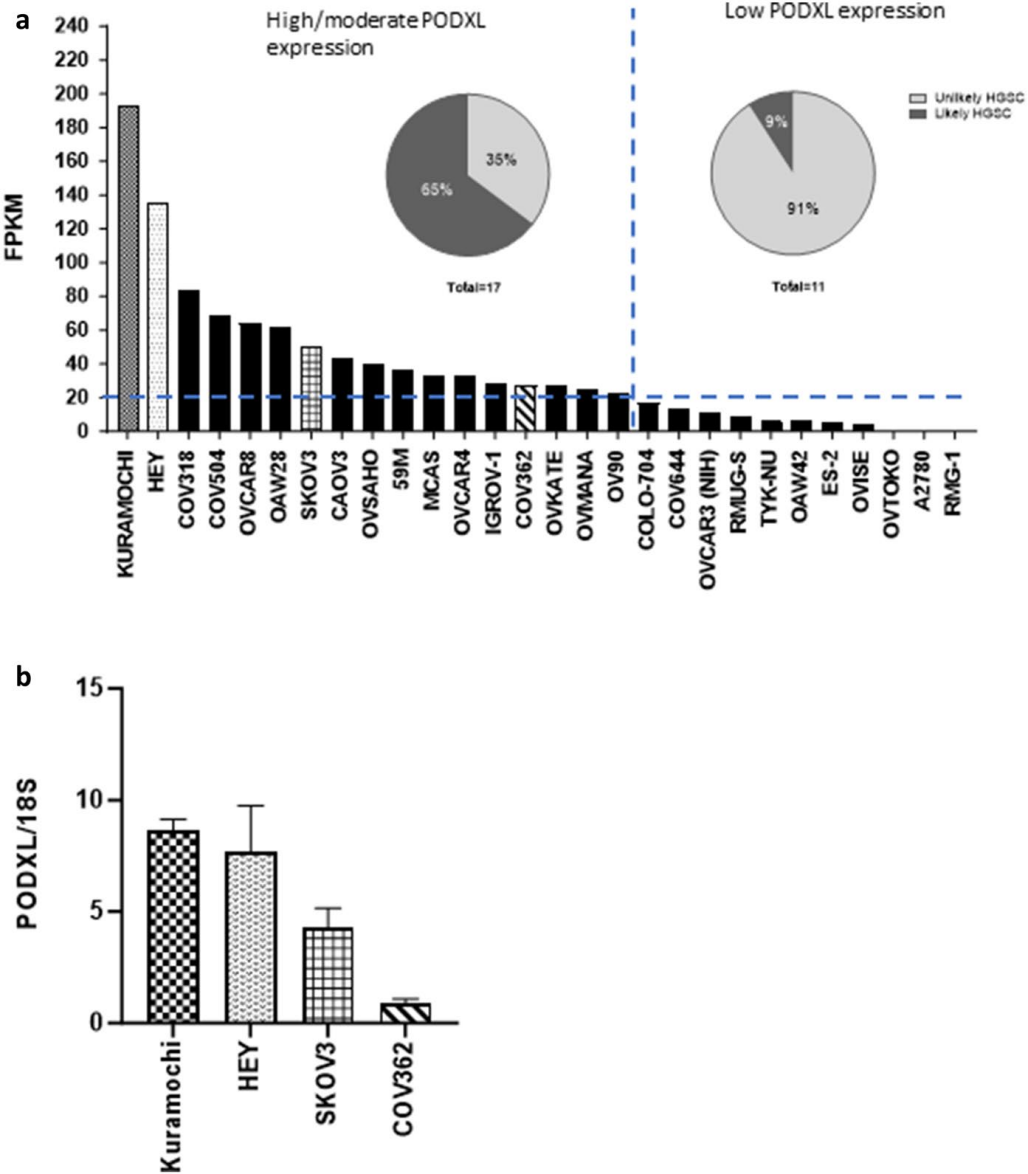
PODXL expression in various human ovarian cancer cell lines. (**A**) Levels of PODXL mRNA in 28 cell lines that were classified as ovarian cancer cells. Data obtained from the European Molecular Biology Laboratory European Bioinformatics Institute and Genentech dataset and expressed in FPKM (fragments per kilobase of transcript per million fragments mapped), < 20 FPKM was deemed low expression. Pie charts, the likelihood of being genetically categorised as HGSC. (**B**) Real-time RT-PCR analysis of PODXL mRNA in 4 representative cell lines shown in (**A**). Data normalized to 18S. Mean ± SD, n = 3.

We next examined how PODXL is expressed in ovarian cancer cell lines. We first searched the EMBL-EBI RNA-seq database and identified 28 ovarian cancer cell lines with PODXL mRNA data available (Fig. 2A). Because PODXL expression was variable, these lines were ordered according to PODXL levels from the highest to lowest, then divided into high/moderate or low groups using 20 FPKM as a cut off (Fig. 2A). These two groups were next analysed separately for their likelihood of being categorised as HGSC, as previously reported by Domcke et al., basing on their genomic profile resemblance to tumour samples^31^. Within the high/moderate PODXL expressing group, 65% (11/17) were likely HGSC; in contrast, only 9% (1/11) of low PODXL expressing lines were likely HGSC (Fig. 2A pie charts). This suggests that high expression of PODXL is more likely associated with HGSC.

**Figure 3:**
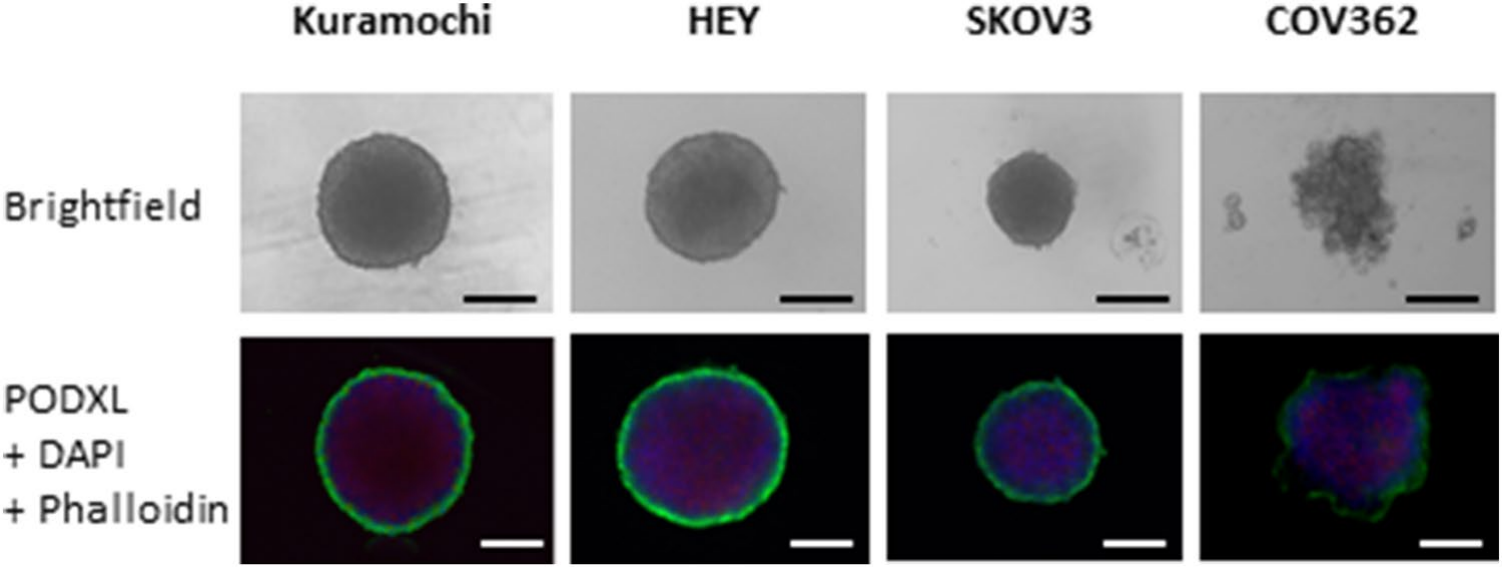
Analysis of spheroids formed with representative ovarian cancer cell lines. The four cell lines shown in Fig. 2B (Kuramochi, HEY, SKOV3 and COV362) were analysed. Bright field images and immunofluorescent staining for PODXL (green), nuclei (blue) and F-actin (red) are shown at 20× magnification. Scale bar: 100 µm.

We next selected four lines (Kuramochi, HEY, SKOV3 and COV362) as representatives and analysed their PODXL expression by RT-PCR (Fig. 2B). Among the four, Kuramochi had the highest whereas COV362 the lowest levels of PODXL mRNA with the other two in between (Fig. 2B), mirroring the trend shown in Fig. 2A, validating the RNA-seq data.

### PODXL is localised to the surface of spheroids formed with ovarian cancer cell lines

The four cell lines (Kuramochi, HEY, SKOV3 and COV362) shown in Fig. 2B were cultured in 96-well ultra-low attachment plates for 72 h to form spheroids. Although they all started with equal numbers of cells, they varied visibly in size and morphology (Fig. 3). Spheroids of Kuramochi and HEY appeared to be large and compact, those of SKOV3 and COV362 were much smaller. Moreover, COV362 formed loose aggregates rather than regular shaped spheroids (Fig. 3).

These spheroids were also examined by immunofluorescence for PODXL and co-stained with phalloidin for F-actin and DAPI for nuclei. The levels of PODXL immunostaining were highest in spheroids of Kuramochi, followed by HEY and SKOV3, whilst spheroids of COV362 had minimal staining (Fig. 3); these results are consistent with the RT-PCR data shown in Fig. 2B. Furthermore, PODXL was localised to the spheroid surface of all cell lines.

### Silencing of PODXL expression in Kuramochi cells

**Figure 4:**
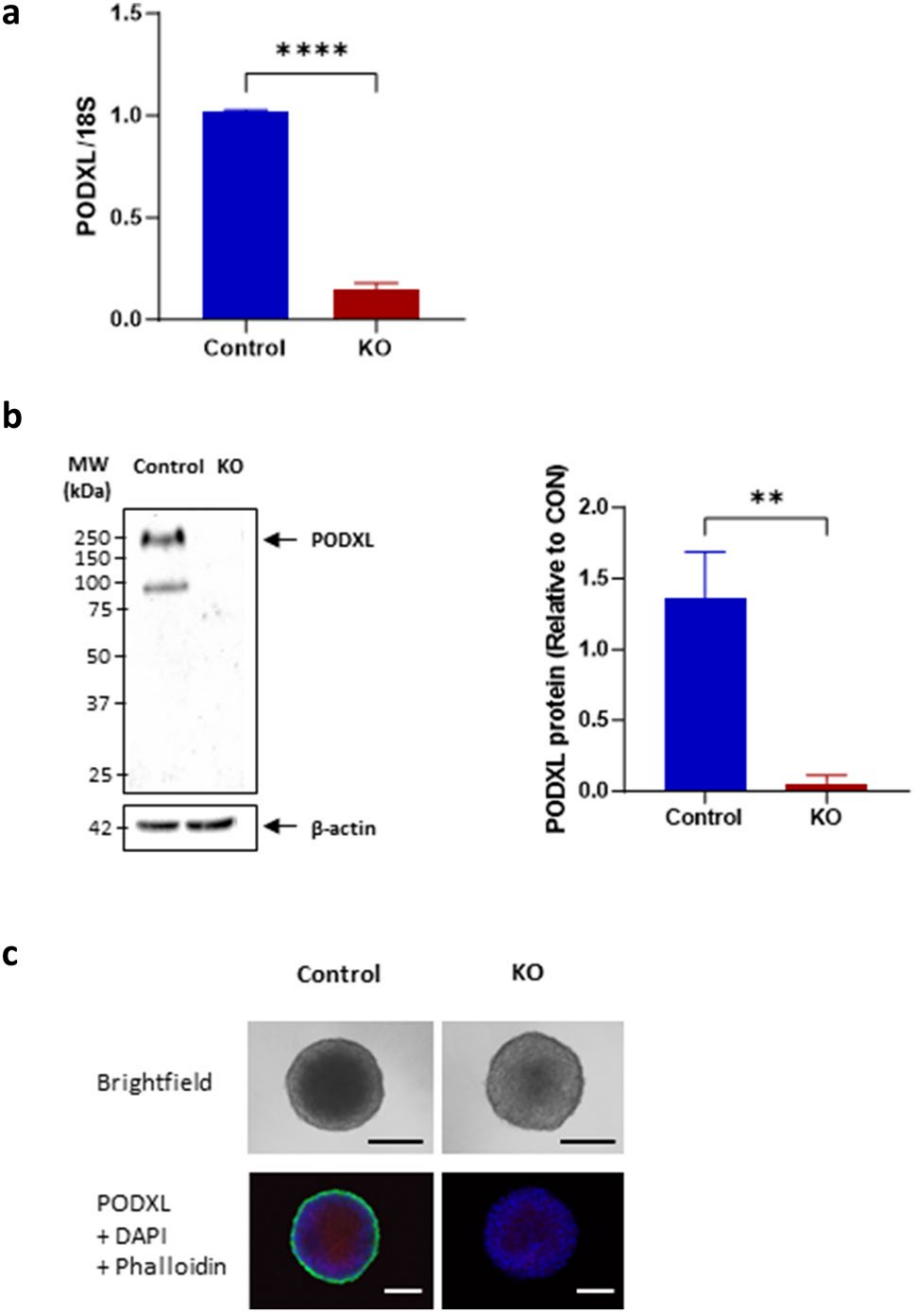
Validation of PODXL-KO in Kuramochi cell line. PODXL was knocked out in Kuramochi and controls were transfected with an empty vector. (**A**) Real-time RT-PCR analysis of PODXL mRNA. Data normalized to 18S and expressed as fold changes relative to control. (**B**) Western blot analysis of PODXL protein, β-actin used as a loading control. Left panel: a representative image. Right panel: densitometric analysis of the top band of PODXL, data normalized to β-actin and expressed as fold changes relative to control (full western blot is presented in Supplementary Fig. 1). (**C**) Representative images of spheroids formed with control and PODXL-KO cells. Top: bright field. Images at 4× magnification. Bottom: immunofluorescent staining of PODXL (green), nuclei (blue), and F-actin (red). Images at 20× magnification. Scale bar: 100 µm. Mean ± SD, n = 3. **P < 0.005, ****P < 0.0001.

Because the Kuramochi cell line showed the highest level of PODXL expression (Figs. 2 and 3), and it mostly resembles HGSC^31^, this line was chosen for further study. To investigate the role of PODXL in spheroid characteristics, PODXL was stably knocked out in Kuramochi cells along with a control that was transfected with an empty vector. PODXL mRNA was readily detected in controls, but the levels were minimal in PODXL-KO cells (Fig. 4A). This was further confirmed by Western blot analysis whereby PODXL protein was detected clearly in controls but below detection in PODXL-KO cells (Fig. 4B). PODXL was also examined by immunocytochemistry in spheroids formed with these cells, positive staining was detected only in spheroids of control but not of PODXL-KO cells (Fig. 4C), further confirming the successful silencing of PODXL expression in PODXL-KO cells.

### PODXL-KO Kuramochi spheroids grow slower, are less compact and more fragile than controls

**Figure 5:**
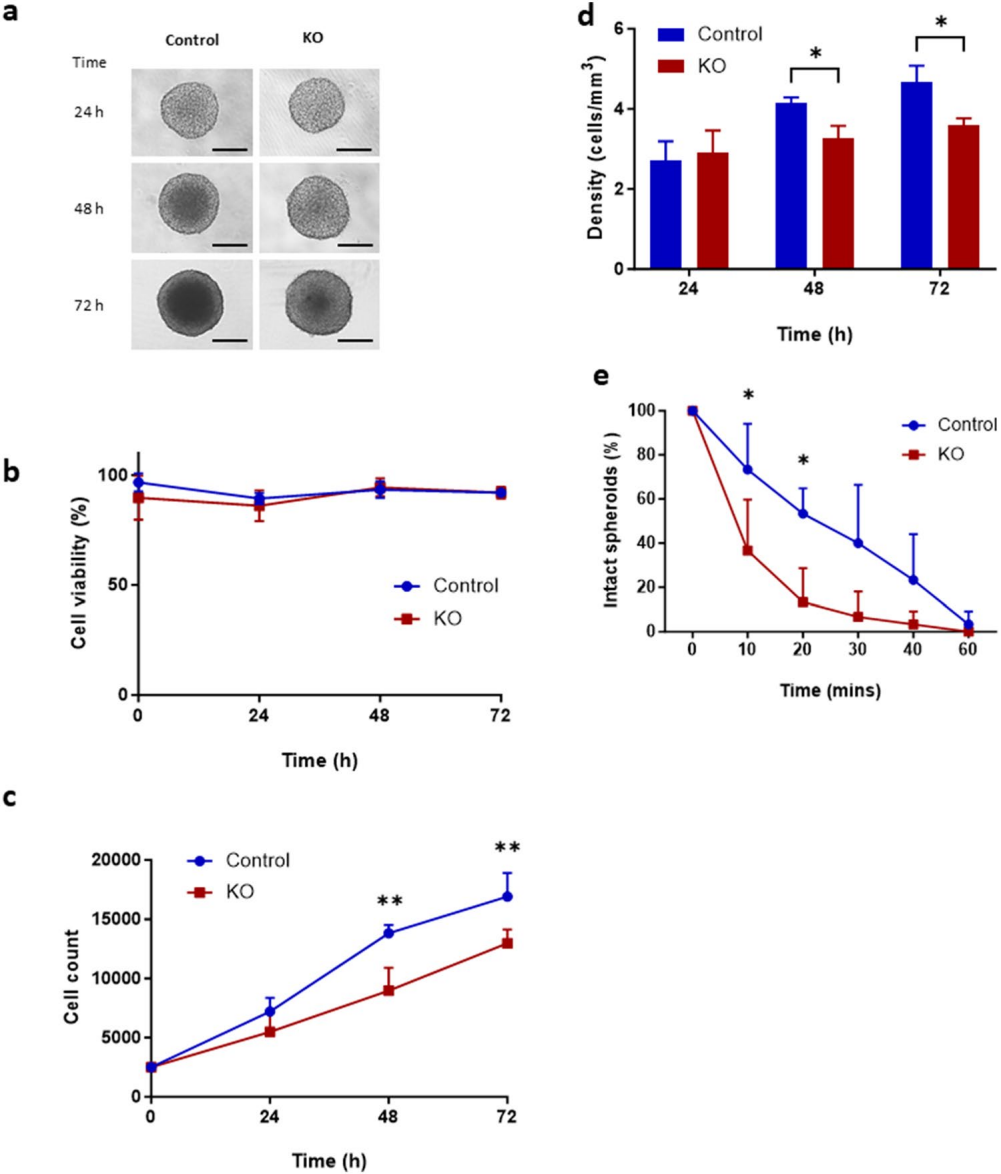
Analysis of growth and spheroid compactness of control and PODXL-KO Kuramochi cells. Control and PODXL-KO spheroids were monitored for 24, 48 and 72 h. (**A**) Representative brightfield images at 4× magnification. Scale bar: 50 µm. (**B**) Cell viability. (**C**) Total number of live cells. (**D**) Spheroid compactness. (**E**) Spheroid hardiness. Data expressed as percentage of intact spheroids over total number of spheroids. Mean ± SD, n = 3. *P < 0.05, **P < 0.005.

We next compared spheroids formed with control and PODXL-KO Kuramochi cells. Because early data of different cell lines indicated an inverse correlation between PODXL expression levels and spheroid size (Fig. 3), we examined whether PODXL-KO affects spheroid size. When equal numbers of control and PODXL-KO cells were cultured for 72 h to form spheroids, no obvious size difference was observed (Fig. 5A). When they were monitored for a further 72 h, spheroid size still did not differ at any time point examined (data not shown). However, when they were dissociated into single cells, while the viability of cells within the spheroids was similar between the two groups (Fig. 5B), PODXL-KO spheroids contained significantly fewer cells compared to the control over time (Fig. 5C). Thus, cell density (calculated as cell number/volume) of PODXL-KO spheroids was also significantly lower than that of controls at both 48 and 72 h (Fig. 5D).

The above observation prompted us to test the frangibility of these spheroids to physical friction. Spheroids from each group were mixed with small steel beads on a plate shaker, and spheroids that remained intact were counted after 10, 20, 30, 40 and 60 min. While all spheroids from both groups were broken apart by 60 min, PODXL-KO spheroids fragmented much faster than controls (Fig. 5E). At 10 min, 70% of control spheroids but only 30% of PODXL-KO spheroids were intact. By 20 min, 50% of control spheroids but only 10% of PODXL-spheroids remained intact (Fig. 5E). Thus, PODXL-KO spheroids were less compact and more fragile than controls.

### PODXL-KO Kuramochi spheroids are more sensitive to carboplatin than controls

**Figure 6:**
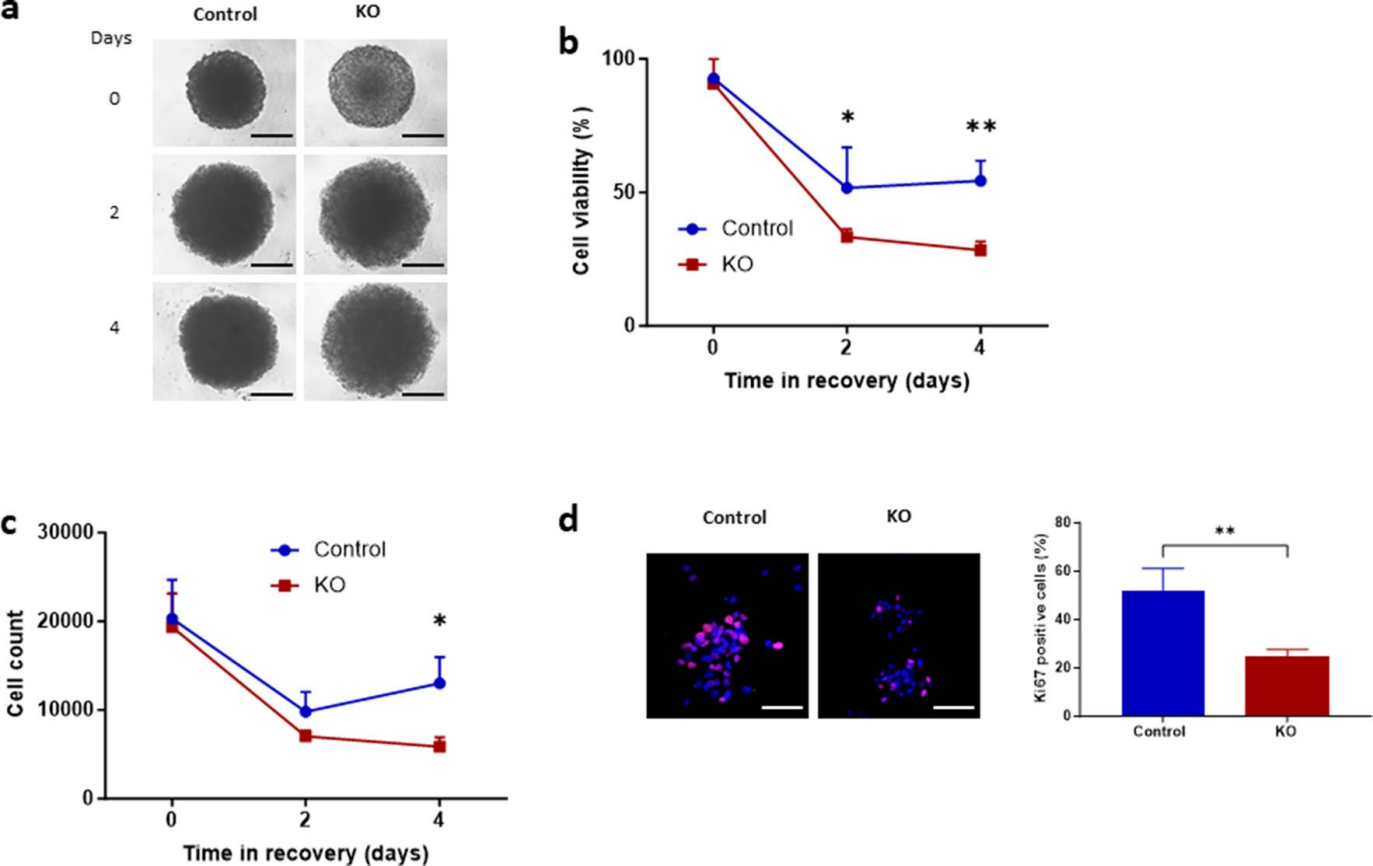
Comparison of control and PODXL-KO Kuramochi spheroids following treatment with carboplatin. (**A**–**C**) Control and PODXL-KO spheroids were treated with carboplatin for 24 h, then monitored for up to 4 days. (**A**) Representative brightfield images of carboplatin treated spheroids in recovery. Scale bar: 100 µm. (**B**) Cell viability. (**C**) Total number of live cells within each spheroid. (**D**) Immunofluorescent staining of Ki-67 in spheroids at day 0 (immediately after carboplatin treatment). Left panel: representative images at 40× magnification. Scale bar: 50 µm. Right panel: percentage of Ki-67-positive cells Mean ± SD, n = 3. *P < 0.05, **P < 0.005.

We next examined the chemosensitivity of the control and PODXL-KO spheroids (Fig. 6). Spheroids were treated for 24 h with 348 µM carboplatin, a mean concentration found in the perfusate of patients undergoing hyperthermic intraperitoneal chemotherapy^32^, then monitored in complete media for up to 4 days to examine post-treatment recovery (Fig. 6A). Immediately after carboplatin treatment, cell viability was similar between PODXL-KO and control spheroids (Fig. 6B). However, during post-treatment recovery, PODXL-KO spheroids decreased in cell viability more quickly than control spheroids and was significantly lower at both day 2 and 4 (Fig. 6B). This difference was further reflected in live cell numbers contained within spheroids (Fig. 6C); while both groups reduced in cell numbers substantially in the initial 2 days, controls started to bounce back from day 2 whereas PODXL-KO spheroids continued to decline. By day 4, the number of live cells contained within control spheroids were double of that in PODXL-KO spheroids (Fig. 6C). When day 0 spheroids were dissociated into single cells and immunostained for cell proliferation marker Ki-67, positive staining was 52% in controls but only 25% in PODXL-KO cells (Fig. 6D), confirming that cells within control spheroids were more actively proliferating than those of PODXL-KO spheroids following treatment (Fig. 6D).

### Ascites-derived primary HGSC spheroids: PODXL expression, spheroid compactness and fragility

**Figure 7:**
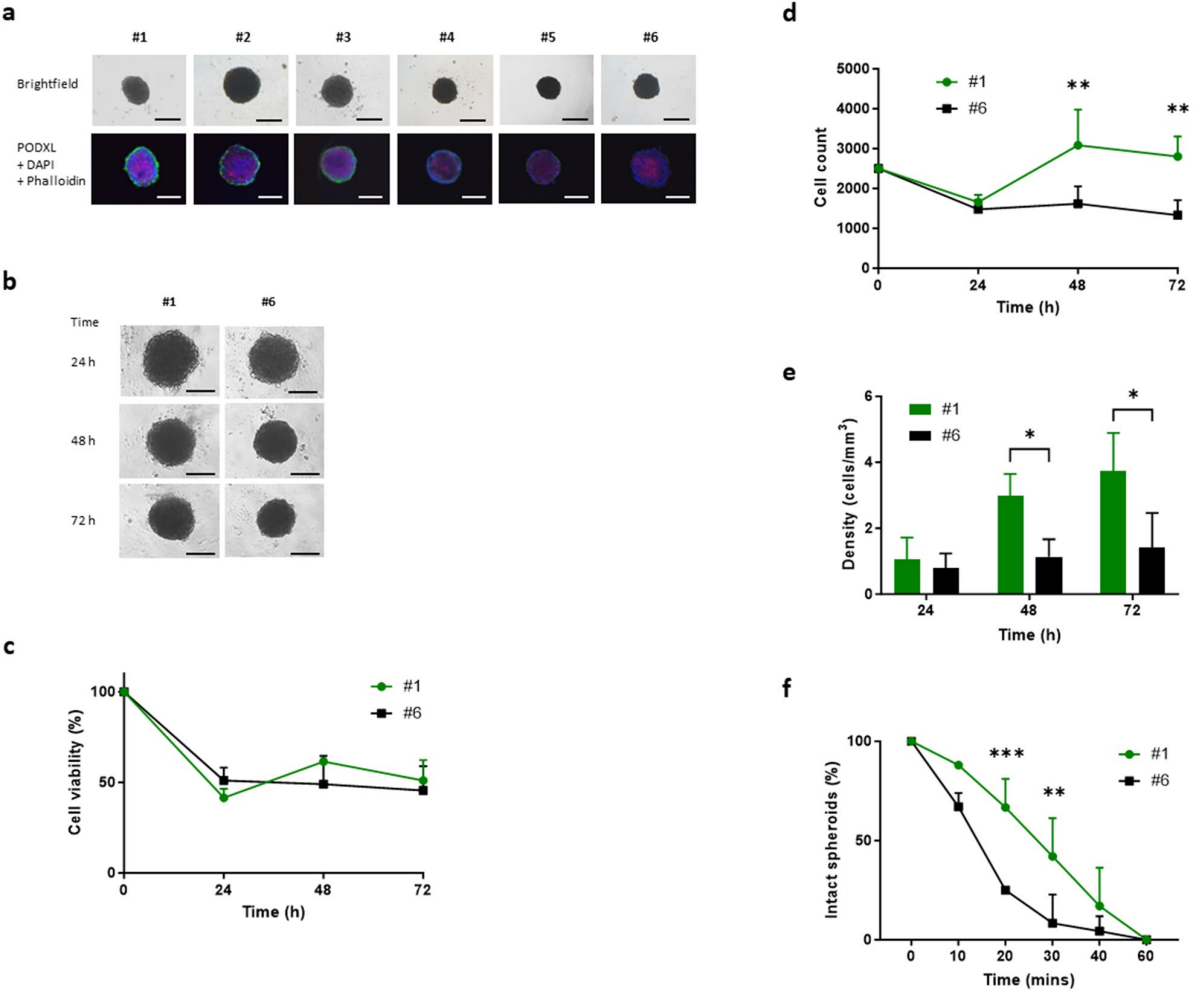
Analysis of cancer spheroids formed with ascites-derived cells from HGSC patients. (**A**) Bright field images of spheroids formed from 6 independent samples examined and their immunofluorescent staining of PODXL (green), nuclei (blue), and F-actin (red). Images at 4 and 20× magnification respectively. (**B**–**F**) Further analysis of cells expressing the highest (#1) and lowest (#6) levels of PODXL. (**B**) Representative brightfield images of spheroids formed over time. Images at 4× magnification. (**C**) Cell viability. (**D**) Total number of live cells. (**E**) Spheroid compactness. (**F**) Spheroid hardiness. Data expressed as percentage of intact spheroids over total number of spheroids. Scale bar: 100 µm. Mean ± SD, n = 3. *P < 0.05, **P < 0.005, ***P < 0.001.

We next investigated spheroids of ascites-derived primary cells obtained from patients with confirmed HGSC (n = 6). All six samples formed spheroids with variations in size and level of PODXL staining, and these were denoted by numbers 1–6 in descending order of PODXL levels (Fig. 7A). The two extremes, #1 and #6, which expressed the highest and lowest levels of PODXL respectively (Fig. 7A), were then chosen for further experiments to determine whether they resemble some of the characteristics of control and PODXL-KO Kuramochi cells respectively.

Spheroids of #1 and #6 primary cells were compared over 72 h for cell viability and numbers (Fig. 7B). While cell viability was similar between the two at each time point examined (Fig. 7C), cell number of #1 spheroids was almost double that of #6 at 48 and 72 h (Fig. 7D). In accordance, cell density of #1 spheroids was also significantly higher than that of #6 at both 48 and 72 h (Fig. 7E). When these spheroids were mixed with small steel beads on a plate shaker, #6 spheroids fragmented much faster than #1 spheroids (Fig. 7F); by 20 min, around 30% of #1 but nearly 70% of #6 spheroids disintegrated (Fig. 7F), by 30 min approximately 60% of #1 but over 90% of #6 spheroids crumbled. All spheroids were broken apart by 60 min. Overall, these results indicate that in spheroids of ascites-derived primary HGSC cells, higher PODXL expression is correlated with the formation of more compact and hardy spheroids, as observed in Kuramochi cell models.

### Ascites-derived primary HGSC spheroids: PODXL levels and spheroid sensitivity to carboplatin

**Figure 8:**
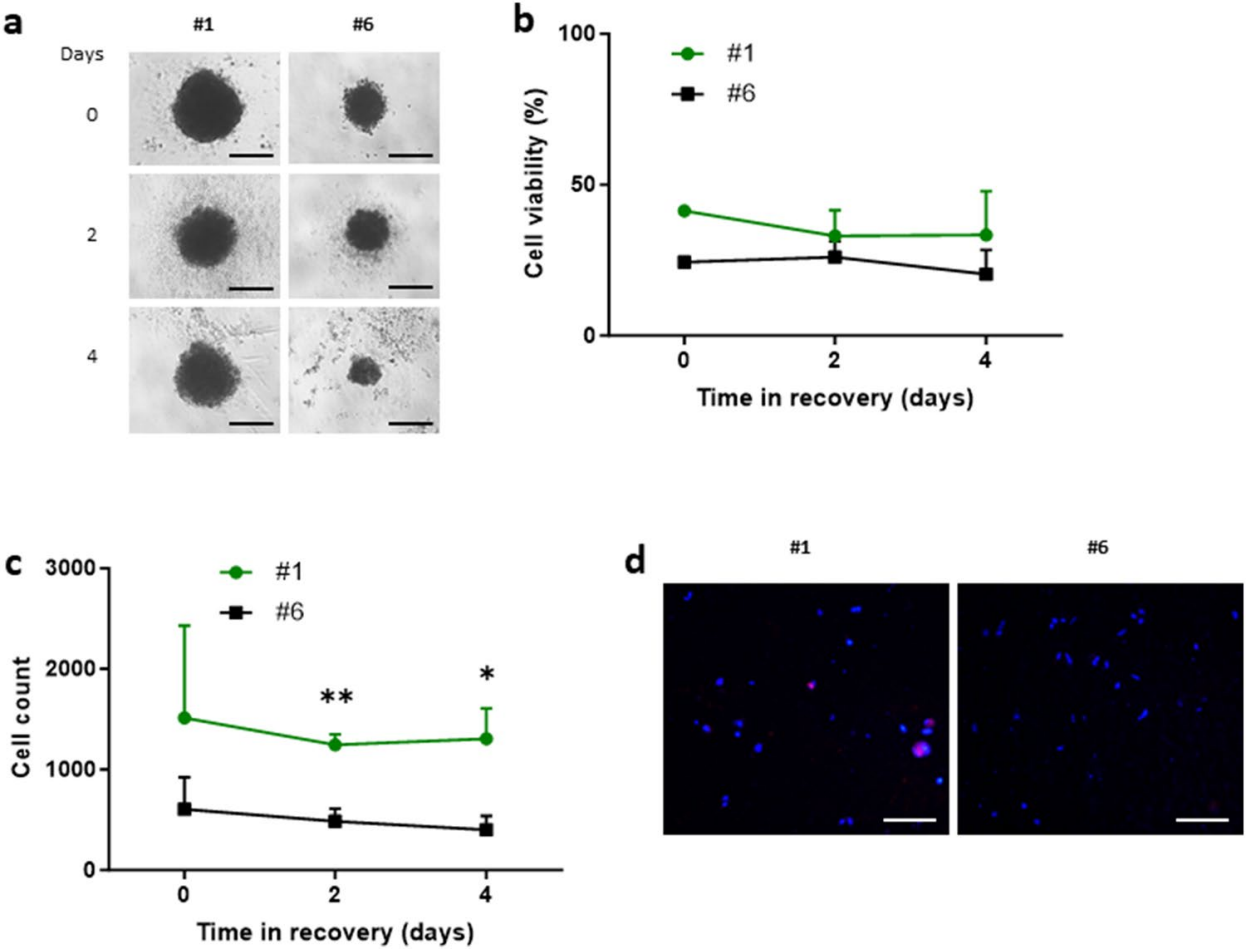
Analysis of cancer spheroids of primary HGSC cells following treatment with carboplatin. Cells expressing the highest (#1) and lowest (#6) levels of PODXL shown in Fig. 7A were analysed. (**A**–**C**) Spheroids were treated with carboplatin for 24 h then monitored for up to 4 days. (**A**) Representative brightfield images of carboplatin treated spheroids in recovery. Images at 4× magnification. (**B**) Cell viability. (**C**) Total Number of live cells within each spheroid. (**D**) Immunofluorescent staining of Ki-67 in spheroids at day 0 (immediately after carboplatin treatment). Representative images at 20× magnification. Scale bar: 100 µm. Mean ± SD, n = 3. *P < 0.05, **P < 0.005.

We next assessed sensitivity of #1 and #6 ascites-derived spheroids to carboplatin. Spheroids were treated with carboplatin for 24 h, then transferred to complete media and their post-treatment recovery was examined (Fig. 8A). While cell viability did not differ significantly between the two (Fig. 8B), there was significantly more live cells within #1 spheroids compared to that of #6 at both days 2 and 4 (Fig. 8C). When these spheroids were dissociated into single cells and stained for Ki-67, some positive staining was observed in #1 whereas staining was absent in #6 cells (Fig. 8D), suggesting cells within spheroids of #1 but unlikely of #6 had proliferative activity. These results indicate an inverse correlation between PODXL levels and sensitivity to carboplatin, which was again, consistent with data of Kuramochi cell models.

## Discussion

HGSC is a lethal gynaecological malignancy that is characterised by intraperitoneal metastasis of cancer spheroids that may persist in ascites after chemotherapy, contributing to disease recurrence and chemoresistance. However, molecular mechanisms are poorly understood. In this study, we examined the presentation of PODXL in HGSC patient tissues and cell lines, and investigated its potential role in promoting cancer spheroid characteristics that may confer survival advantage. PODXL was immunostained in all HGSC patient tissues examined (n = 17) with variable intensities. PODXL was also expressed by most ovarian cancer cell lines examined (n = 28), and those with high PODXL expression are more likely categorised as HGSC. Using the cell line expressing the highest levels of PODXL as a model and CRISPR/Cas9 gene editing, we further illustrated that PODXL promoted cell proliferation within the spheroid, and fostered the formation of dense cancer spheroids which were resilient to fragmentation. Furthermore, following treatment with carboplatin, more cells bounced back and started to proliferate within spheroids that express higher PODXL. These characteristics were also observed in primary cancer cells derived from ascites of HGSC patients (n = 6). Our studies thus suggest that PODXL may play an important role in enhancing spheroid survival against physical friction and chemotherapy drugs.

All HGSC tumour tissues examined here (n = 17) were positively immunostained for PODXL although the levels varied. This supports previous findings that PODXL is expressed in an overwhelming number of HGSC cases^26^. Furthermore, in tissues examined here, 29% showed intense and membranous PODXL staining, localising around the outer layer of cancer clusters, which resemble the morphology of budding tumors. These findings are consistent with prior studies in ovarian, colorectal, urothelial bladder, pancreatic and breast cancer, in which apical surface expression of PODXL on tumor cells was associated with poor prognosis^26,27,33–36^. Previous studies in colorectal cancer also found distinct membranous staining of PODXL predominantly in a subset of infiltrating cells at the tumour front, which led to the suggestion that overexpression of PODXL is associated with invasive and metastatic properties^33,37,38^. Studies of MCF-7 breast cancer cells in mice further showed that PODXL overexpression promotes budding of cancer clusters from the primary tumors^27^. Previous studies also reported an association between surface PODXL expression and shorter disease-free survival in HGSC patients^26^. However, we were unable to ascertain this association in our cohort due to the lack of full patient prognosis data.

In ascites, spheroids can exist in different sizes and morphology, ranging from loose cellular aggregates to tight spheroids, and compact spheroids were reported to confer a survival advantage^8,39,40^. Here, we demonstrated that PODXL is expressed on the surface of both established ovarian cancer cell lines and patient ascites-derived spheroids, and that PODXL levels positively correlated with spheroid compactness. Kuramochi, a HGSC cell line confirmed by its genomic profile^31^, showed the highest levels of PODXL expression and formed compact spheroid, whereas COV362 which expressed much lower PODXL only formed loose cellular aggregates. When PODXL is knocked out in Kuramochi, while spheroid size was not altered, the control spheroids contained significantly more cells over time than that of PODXL-KO cells; because cell viability did not differ between the two groups, this difference in cell number suggest that proliferation was higher within the spheroids of control than PODXL-KO cells. Consequently, control spheroids were significantly more compact than PODXL-KO. Similarly, in ascites-derived primary spheroids, those with higher PODXL expression were denser than spheroids of lower PODXL.

We thus next investigated whether the above differences relate to spheroid fragility, by establishing a novel technique of mixing spheroids with steel beads on a rocking platform. To our best knowledge, this is the first report of such experimentation. PODXL-KO Kuramochi spheroids disintegrated much faster than control spheroids. Likewise, primary spheroids with lower PODXL levels exhibited significantly higher rates of fragmentation compared to those with higher PODXL expression. All spheroids were broken apart by 60 min, indicating a maximum threshold was reached. While there are currently no reports on the correlation between spheroid compactness and tumorigenesis, we demonstrated here a positive link between PODXL expression, spheroid compactness and hardiness, which may confer advantages for their survival in ascites.

Combined with tumour debulking, the first-line treatment for ovarian cancer consists of a platinum agent carboplatin and a taxane paclitaxel, which selectively eliminates highly proliferative cells^41,42^. While most HGSC patients will respond to initial treatment, more than 80% will have a reoccurrence^42^, and cancer spheroid evasion of chemotherapy is believed to be a contributor. By mimicking the multicellular structures present in in vivo, 3D spheroid models in vitro provide a good system to study chemoresistance and gene expression^43–46^. PODXL has been shown to significantly mediate cisplatin resistance in oral tongue squamous carcinoma and osteosarcoma cells^29,47^. In this study, we showed that control spheroids were more resilient to carboplatin (an analogue of cisplatin) than PODXL-KO spheroids, with the former containing significantly higher number of live cells over time following treatment. This was reflected by a significantly higher percentage of Ki-67-positive cells present within the control compared to the PODXL-KO Kuramochi spheroids, consistent with PODXL promoting cell proliferation. A similar observation was also made in primary spheroids following treatment with carboplatin, those with high PODXL levels showed higher cell number and Ki-67 expression than spheroids of low PODXL expression.

We have previously reported that PODXL promotes an anti-adhesive and impermeable epithelial barrier^48^. This mechanism may be involved in HGSC spheroids where PODXL expression likely hinders drug penetration into the spheroid, but further studies are needed to confirm this speculation. A limitation of this study was the small cohort of HGSC patients and ascites-derived primary cells that we were able to acquire. In future studies, analysis of a larger cohort of patients will provide a more comprehensive view of the importance of PODXL in primary spheroids. Moreover, given the heterogeneous nature of HGSC, there may be other factors/mechanisms that may interact with PODXL to support proliferation and survival of cancer spheroids.

In summary, our results suggest that PODXL plays an important role in facilitating the formation of compact and hardy spheroids, which are resistant to physical fragmentation and resilient to chemotherapy drugs. Future studies are needed to elucidate the exact mechanisms by which PODXL confers these survival advantages in HGSC spheroids.

## Materials and methods

### Patient samples

Primary HGSC tumour tissue biopsies (n = 17) plus one benign ovarian sample and ascites (n = 6) were acquired from patients admitted to Eastern Health hospitals in Melbourne, Australia; each patient provided written informed consent and ethics approval was obtained from Monash University Human Research Ethics Committee (#06032C). All procedures were conducted in accordance with the relevant guidelines and regulations. Tissues were fixed in 100% ethanol and processed into paraffin blocks. Non-adherent cancer cells in ascites fluid were isolated using an established purification method^8^. Histopathology and clinical information for each sample was obtained from de-identified pathology reports.

### Immunohistochemistry

The procedure was performed at room temperature unless specified. Paraffin sections (5 µm thickness) were deparaffinised in histosol (ChemSupply, SA, AUS), rehydrated, then microwaved in pre-boiled 0.01 M citrate buffer (pH 6.0) for antigen retrieval. Endogenous peroxidase activity was blocked for 10 min with 3% H_2_O_2_ in methanol, tissues were then incubated for 20 min with 15% horse serum in high salt TBS (0.3268 M NaCl, 0.05 M Tris base pH 7.6) containing 0.1% Tween20 (Sigma-Aldrich, MO, USA). Sections were next incubated for 1 h at 37 °C with mouse monoclonal antibodies against human PODXL (2 µg/ml, Santa Cruz biotechnology, #SC-23904) or mouse IgG (negative control, Dako, Glostrup, Denmark, #X0931). Subsequently tissues were incubated with biotinylated horse-anti-mouse IgG (Vector laboratories, CA, USA, #BA-2000) for 30 min, and then with avidin–biotin complex conjugated to horseradish peroxidase (Vector laboratories #PK-6100) for 30 min. Between incubations, sections were washed with high salt TBS containing 0.6% Tween20. Colour was developed with peroxidase substrate 3/3′-diaminobenzidine (DAB) (Dako, #K3466), and sections were counter-stained with Harris haematoxylin (Sigma-Aldrich #HHS16) and mounted with DPX reagent (Sigma-Aldrich #06522). Slides were scanned at 40X magnification using Aperio Scanscope XT (Leica Biosystems Pty Ltd, Wetzlar, Germany), and analysed using the Aperio ImageScope (v12.1.0.5029) software (Leica Biosystems Pty Ltd).

### Bioinformatic analysis of PODXL expression in ovarian cancer cell lines

RNA-seq data were obtained from EMBL-EBI (European Molecular Biology Laboratory's European Bioinformatics Institute European Bioinformatics Institute) database (https://www.ebi.ac.uk), and 37 ovarian cancer cell lines with varying levels of PODXL expression were identified, of which 28 were further analysed after those with ambiguous or previously misidentified origins were excluded.

### Cell culture

The Kuramochi cell line, representing HGSC with the highest PODXL expression, was purchased from JCRB CellBank Australia (#JCRB0098). The study also examined another 3 commonly used epithelial ovarian cancer cell lines HEY, SKOV3 and COV362^49^, which were all authenticated at the Hudson Institute of Medical Research (Clayton, VIC, Australia). Kuramochi and HEY cells were maintained in RPMI 1640 Medium + GlutaMAX supplement (Thermo Fisher Scientific, MA, USA, #61870036), whereas SKOV3 was maintained in DMEM/F-12 + GlutaMAX supplement (Thermo Fisher Scientific #10565018) and COV362 in DMEM (Thermo Fisher Scientific, #11965092). Primary ascites-derived HGSC cells were isolated as reported previously^8^, and were maintained in a 1:1 ratio of Medium 199 (Thermo Fisher Scientific, #11150-059) and MCDB131 (Thermo Fisher Scientific, #10372-019). All media were supplemented with 10% (15% for primary cells) fetal bovine serum (FBS, Thermo Fisher Scientific) 1% antibiotic–antimycotic (Thermo Fisher Scientific, #15240062), and cells were cultured at 37 °C under 5% CO_2_.

### Silencing PODXL expression

PODXL expression was silenced using CRISPR/Cas9 gene editing with one single guide RNA sequence (TAGATGAGTCCGTAGTAGTC) targeting exon 2 region of PODXL, as per the Zhang lab protocol^50^, and the empty vector was used as the control. Kuramochi cells were transfected with Lipofectamine 2000 (Thermo Fisher Scientific #11668019) mixed with plasmid (3:1 ratio) in Opti-MEM (Thermo Fisher Scientific #51985034) for 24 h, then cultured in complete growth medium containing 1 µg/ml puromycin (Sigma-Aldrich, #P8833). When reaching 80% confluency, cells were trypsinized, seeded sparsely in 10 cm dishes (2–5000 cells/dish), and cultured until colonies formed. Individual colonies were then expanded sequentially in 48-, 24-, 12-, and 6-well plates, then in T25 and T75 flasks. PODXL-KO cells were confirmed by real time RT-PCR, Western blot analysis and immunocytochemistry.

### RNA extraction and real-time RT-PCR analysis

Total RNA was extracted with TRI reagent (Sigma-Aldrich, #93289) for cell lines and RNeasy Mini Kit (Qiagen, Hilden, Germany, #74104) for primary cells. RNA concentration was determined by Nanodrop (Thermo Fisher Scientific), and 500 ng total RNA was reverse transcribed into complementary DNA (cDNA) using SuperScript III First-Strand kit (Invitrogen #18080-051) as per the manufacturer’s protocol. RT-PCR was performed using SYBR Green Master Mix (Thermo Fisher Scientific, #A25742) on the Quantstudio 7 Flex System (Thermo Fisher Scientific) with the following conditions: (1) 95 °C for 10 min, (2) 40 cycles of denaturation (95 °C for 15 s), annealing (60 °C for 40 s) and extension (95 °C for 15 s), and (3) dissociation curve assessment (60–95 °C). The following primers were used: PODXL (forward 5′ GAGCAGTCAAAGCCACCTTC 3′, reverse 5′ GCTGGAATTACCGCGGCT 3′) and 18S (forward 5′ CGGCTACCACATCCAAGGAA 3′, reverse 5′ GCTGGAATTACCGCGGCT 3′). PODXL mRNA was normalised against 18S and the relative levels were calculated as fold changes using ΔΔCT. All experiments were performed 3 times independently.

### Western blot analysis

Cells were lysed in buffer containing 50 mM Tris, 150 mM NaCl, 2 mM EDTA, 25 mM NaF (serine/threonine protein phosphatase inhibitor), 0.2% Triton X-100, 0.3% Nonidet P-40 (Millipore, Sigma-Aldrich), 25 mM glycerolphosphate (a phosphatase inhibitor) with the addition of a complete protease inhibitor cocktail (Roche Molecular, Mannheim, Germany). Lysates underwent 3 times of freeze–thaw processes (10 min dry ice, 5 min room temperature and 5 min ice) before being centrifuged at 14,000 rpm for 10 min at 4 °C. Proteins were separated on a 10% Polyacrylamide SDS-PAGE gel and transferred to PVDF membranes (GE Healthcare, NSW, Australia). After blocking for 5 h at room temperature with 5% BSA (Bovogen, VIC, Australia) in Tris-buffered saline [10 mmol/L Tris (pH 7.5) 232 and 0.14 mol/L NaCl] containing 0.2% Tween20 (TBST-T), the membrane was incubated overnight at 4 °C with polyclonal goat antibodies against human PODXL (2.5 µg/ml in PBS-T, R&D Systems, MN, USA #AF1568), then 1 h with a rabbit anti-goat IgG-HRP (diluted 1:2000 in TBS-T; Dako, USA #P0449). Bands were visualised using Lumi-Light Western Blotting Substrate (Roche Molecular, #12015200001) and ChemiDoc MP Imaging System (Bio-Rad, CA, USA). Membranes were subsequently probed for β-actin as a loading control using an HRP-conjugated β-actin antibody (2 µg/ml, Cell Signalling Technology, MA, USA). All experiments were repeated 3 times independently.

### Cancer spheroids formation and analysis

Spheroids were formed by culturing 2500 cells per well in 96-well round bottomed ultra-low attachment plate (Merck, Darmstadt, Germany, #CLS7007-24EA), and individual spheroid was collected after 72 h by manual pipetting. Spheroids were monitored using a Nikon eclipse TS100 microscope equipped with a Nikon DS-Fi1 camera (Tokyo, Japan), and images were acquired using the Nikon digital sight DS-L2 unit. To determine the number and viability of cells within spheroids, individual spheroid was trypsinised and dissociated into single cells and analysed using an automated cell counter Countess 3 (Thermo Fisher Scientific). To calculate cell density of spheroids, the diameter of each individual spheroid was determined prior to cell dissociation by averaging the length of 4 different angles using the “straight line” function on the ImageJ software version 1.53c (NIH, USA). The average spheroid volume was then calculated using the formula $4/3\pi {r}^{3}$, and density was estimated as total live cell number/volume. To test spheroid fragility, 10 spheroids were transferred into a 24-well plate containing 5 stainless steel beads (5 mm in diameter, Sigma Aldrich, #BMSD113450) containing complete media. The plate was then placed on a shaker (140 RPM), and spheroids that still remained intact were counted after 10, 20, 30, 40 and 60 min; any spheroid with visible fragmentation was considered not intact. All experiments were repeated 3 times independently.

### Immunofluorescence

All procedures were performed at room temperature unless stated otherwise. Spheroids were fixed with 4% paraformaldehyde in PBS for 30 min, permeabilised with 0.1% Triton X-100 in PBS for 10 min, and blocked with 15% normal donkey serum in 1% BSA/PBS for 2 h. Spheroids were incubated overnight at 4 °C with goat anti-human PODXL antibodies (2.5 µg/ml diluted in 1% BSA/PBS, R&D Systems, #AF1568) or goat IgG (2.5 µg/ml diluted in 1% BSA/PBS, R&D Systems, #AB-108-C), then 2 h with Alexa Fluor 488 donkey anti-goat antibodies (1 µg/ml diluted in 1% BSA/PBS, Invitrogen, #A11055). F-actin was subsequently stained with phalloidin 555 (1:40 dilution in PBS, Thermo Fisher Scientific, #A34055) for 30 min, and nuclei counterstained with DAPI (0.5 µg/ml diluted in PBS, R&D Systems, #AB-108-C) for 10 min. For imaging, 10 mm glass coverslips (Marienfeld, Laidao-Königshofen, Germany, #0111500) were mounted on the corners of the microscope slide (Marienfeld, #0705002) to function as spacers. Spheroids were then pipetted to the middle of the slide with a drop of fluorescent mounting agent (Dako, #S3023), mounted with a coverslip, and analysed using the Nikon ECLIPSE Ti A1R confocal microscope (RMIT University).

To assess Ki-67 positive cells, spheroids were resuspended into single cells in TrypLE (Thermo Fisher, #12604021), pipetted onto a droplet of Histogel (Epredia, MI, USA, #HG-4000-012), then smeared onto a microscope slide. After the gel was air dried, cells were fixed and immunostained as described above but with anti-Ki-67 rabbit antibody (1:250 dilution in 1% BSA/PBS, Abcam, Cambridge, UK, #ab16667) or rabbit IgG (4 μg/ml diluted in 1% BSA/PBS, Dako, #X0936), and a rabbit-anti mouse Alexa Fluor 568 antibody (1:200 dilution in 1% BSA/PBS, Thermo Fisher Scientific, #a10042). All experiments were repeated 3 times independently.

### Treatment of cancer spheroids with carboplatin

Spheroids were treated with a clinical dose of 348 µg/ml carboplatin (Hospira, IL, USA)^32^ for 24 h then maintained in complete media and analysed at day 2 and day 4. Culture media was refreshed every 2 days by replacing half of the culture media with fresh media. All experiments were repeated 3 times independently.

### Statistical analysis

GraphPad Prism version 10 (GraphPad Software, San Diego, CA) was used for statistical analysis. Students *t* test or two-way ANOVA was applied wherever appropriate, and data were expressed as mean ± SD; P ≤ 0.05 was considered significant.

### Supplementary Information

Supplementary Figure 1.

## Data Availability

All data associated with this study are present in the paper or in the supplementary materials.
