# Supplementary material for: Podocalyxin promotes the formation of compact and chemoresistant cancer spheroids in high grade serous carcinoma
Source: Sci Rep. 2024 Mar 30;14:7539. doi: 10.1038/s41598-024-57053-7 (PMC10980795; doi:10.1038/s41598-024-57053-7)
Supplement: Supplementary file 1 — Supplementary Figure 1. [file 41598_2024_57053_MOESM1_ESM.pdf]

Podocalyxin promotes the formation of compact and chemoresistant cancer spheroids in high grade serous carcinoma

Ngoc Le Tran[1], Yao Wang[1], Maree Bilandzic[2,3], Andrew Stephens[2,3], and Guiying Nie[1*]

Affiliations:

[1]Implantation and Pregnancy Research Laboratory, School of Health and Biomedical Sciences, RMIT University, Bundoora, Victoria, 3083, Australia

[2]Hudson Institute of Medical Research, Clayton, Victoria, 3168, Australia

[3]Department of Molecular and Translational Sciences, Monash University, Clayton, Victoria, 3168, Australia

*Corresponding author:

Guiying Nie

ORCID: 0000-0001-9641-087X

Implantation and Pregnancy Research Laboratory,

School of Health and Biomedical Sciences,

RMIT University,

Bundoora West Campus

Victoria, 3083, Australia

Phone: +61 3 9925 7274

Email: guiying.nie@rmit.edu.au

A.

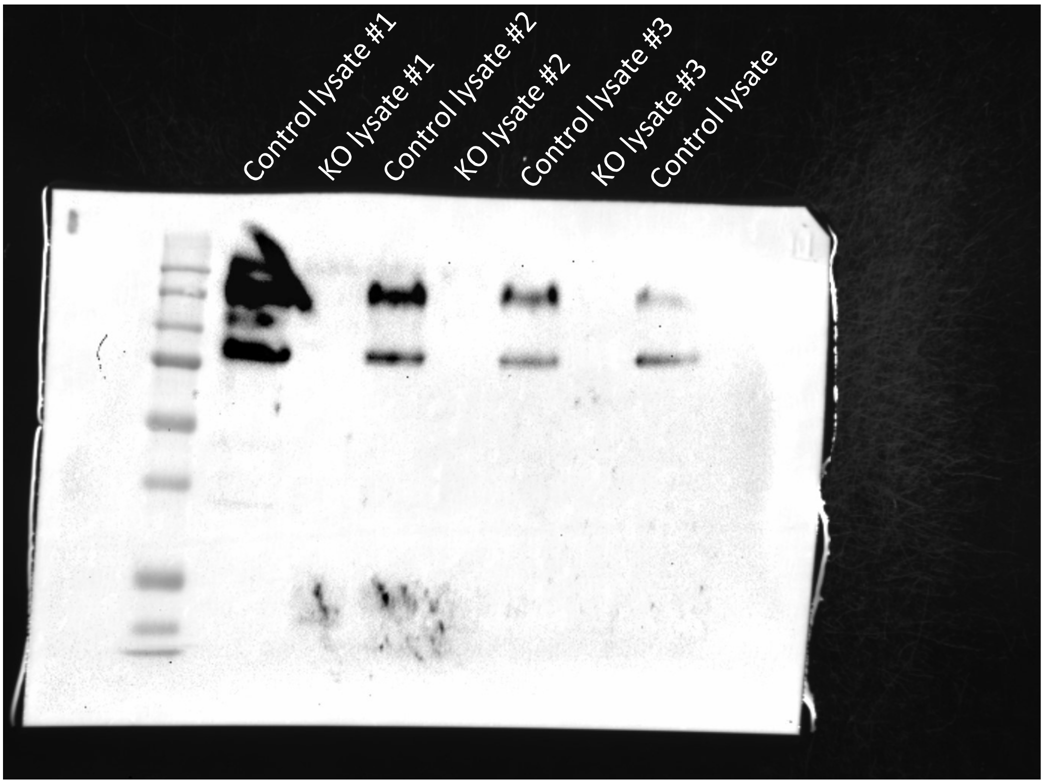

B.

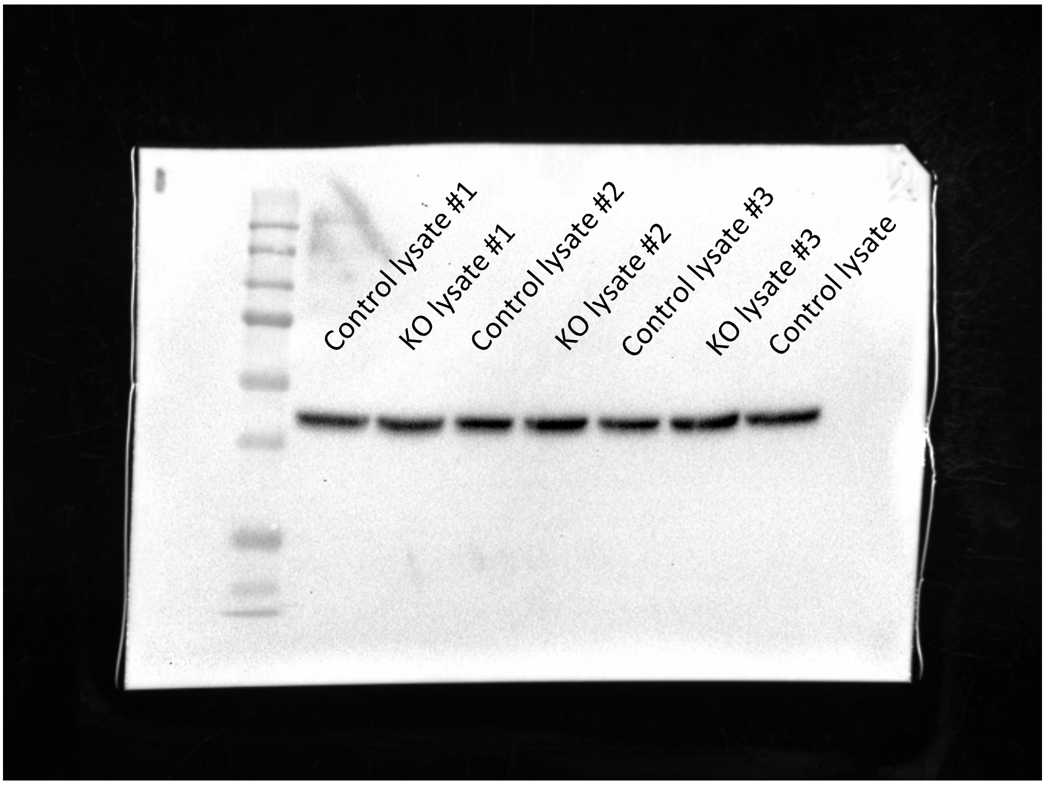

Supplementary Figure 1. Full western blot image of Fig. 4B. A) Full image of human PODXL. B) Full image of β-actin. Representative images used is from control and KO lysate #3.
